# Decoding the dopamine transporter imaging for the differential diagnosis of parkinsonism using deep learning

**DOI:** 10.1007/s00259-022-05804-x

**Published:** 2022-05-19

**Authors:** Yu Zhao, Ping Wu, Jianjun Wu, Matthias Brendel, Jiaying Lu, Jingjie Ge, Chunmeng Tang, Jimin Hong, Qian Xu, Fengtao Liu, Yimin Sun, Zizhao Ju, Huamei Lin, Yihui Guan, Claudio Bassetti, Markus Schwaiger, Sung-Cheng Huang, Axel Rominger, Jian Wang, Chuantao Zuo, Kuangyu Shi

**Affiliations:** 1 PET Center, Huashan Hospital, Fudan University, No 518, East Wuzhong Road, Xuhui District, Shanghai, China; 2 National Center for Neurological Disorders & National Research Center for Aging and Medicine, Huashan Hospital, Fudan University, Shanghai, China; 3 Department of Nuclear Medicine, Inselspital, Bern University Hospital, University of Bern , Bern, Switzerland; 4 Department of Informatics, Technische Universität München, Munich, Germany; 5 AI Lab, Tencent, Shenzhen, China; 6 Department of Neurology, Huashan Hospital, Fudan University, Shanghai, China; 7 Department of Nuclear Medicine, University of Munich, Munich, Germany; 8 Department of Neurology, Inselspital, Bern University Hospital, University of Bern, Bern, Switzerland; 9 Klinikum R. d. Isar, Technische Universität München, Munich, Germany; 10 Department of Molecular & Medical Pharmacology, University of California, Los Angeles, CA USA

**Keywords:** Parkinson’s disease, Dopamine transporter imaging, Atypical parkinsonian syndrome, Differential diagnosis, Deep neural network

## Abstract

**Purpose:**

This work attempts to decode the discriminative information in dopamine transporter (DAT) imaging using deep learning for the differential diagnosis of parkinsonism.

**Methods:**

This study involved 1017 subjects who underwent DAT PET imaging ([^11^C]CFT) including 43 healthy subjects and 974 parkinsonian patients with idiopathic Parkinson’s disease (IPD), multiple system atrophy (MSA) or progressive supranuclear palsy (PSP). We developed a 3D deep convolutional neural network to learn distinguishable DAT features for the differential diagnosis of parkinsonism. A full-gradient saliency map approach was employed to investigate the functional basis related to the decision mechanism of the network. Furthermore, deep-learning-guided radiomics features and quantitative analysis were compared with their conventional counterparts to further interpret the performance of deep learning.

**Results:**

The proposed network achieved area under the curve of 0.953 (sensitivity 87.7%, specificity 93.2%), 0.948 (sensitivity 93.7%, specificity 97.5%), and 0.900 (sensitivity 81.5%, specificity 93.7%) in the cross-validation, together with sensitivity of 90.7%, 84.1%, 78.6% and specificity of 88.4%, 97.5% 93.3% in the blind test for the differential diagnosis of IPD, MSA and PSP, respectively. The saliency map demonstrated the most contributed areas determining the diagnosis located at parkinsonism-related regions, e.g., putamen, caudate and midbrain. The deep-learning-guided binding ratios showed significant differences among IPD, MSA and PSP groups (*P* < 0.001), while the conventional putamen and caudate binding ratios had no significant difference between IPD and MSA (*P* = 0.24 and *P* = 0.30). Furthermore, compared to conventional radiomics features, there existed average above 78.1% more deep-learning-guided radiomics features that had significant differences among IPD, MSA and PSP.

**Conclusion:**

This study suggested the developed deep neural network can decode in-depth information from DAT and showed potential to assist the differential diagnosis of parkinsonism. The functional regions supporting the diagnosis decision were generally consistent with known parkinsonian pathology but provided more specific guidance for feature selection and quantitative analysis.

**Supplementary Information:**

The online version contains supplementary material available at 10.1007/s00259-022-05804-x.

## Introduction

The accurately and timely differential diagnosis of parkinsonian disorders remains challenging due to overlapping symptoms, especially in the early stage, between patients with idiopathic Parkinson’s disease (IPD) and atypical parkinsonian syndromes (APS), e.g., multiple system atrophy (MSA) and progressive supranuclear palsy (PSP) [1]. Pathological examination results show that approximately 20–30% of patients with MSA or PSP are initially misdiagnosed as IPD in clinical practice [1]. Therefore, developing an accurate computer-aided diagnosis method for differential diagnosis of parkinsonian disorders is of great value to avoid unnecessary testing and inappropriate medicines and thus leads to better therapeutic strategies.

The dopamine transporter (DAT) imaging such as [^11^C]CFT positron emission tomography (PET) and [^123^I]FP-CIT single-photon emission computed tomography (SPECT) (DaTscan) can reflect the subject’s dopaminergic degeneration and therefore is a powerful diagnostic tool [2]. Nowadays, striatal DAT quantification together with visual analysis is utilized as a standard practice in clinical studies [3]. However, according to current knowledge, DAT imaging has not been confirmed to be suitable for the reliable differentiation of IPD and APS subtypes based on conventional quantitative analyses such as the DAT binding ratio (BR) quantification [4–6]. These conventional quantitative analyses normally focus on specific brain regions including putamen and caudate, and each region is represented by its mean counts, hence underutilizing the global information of entire DAT scans, especially the distribution of the uptakes within each region as well as correlations of different brain regions.

The deep neural network has been demonstrated to be able to decode in-depth features automatically and effectively from the data [7–11], which has the potential to discover more comprehensive information and update its parameters specifically for the differential diagnosis of parkinsonism. Moreover, deep learning may assist the conventional quantitative analysis or radiomics analysis [12–14]. The potential of deep learning has been revealed in the analysis of DAT imaging [15–18]. Choi et al. introduced the deep learning method to refine the imaging diagnosis of Parkinson's disease based on the FP-CIT SPECT scans[16]. Wenzel et al. reported that the deep neural network can be trained to be robust to variable image characteristics for the classification of the FP-CIT SPECT [17]. Currently, these works mainly focused on the differentiation between PD and healthy controls but did not evaluate the potential of deep learning to solve a more challenging task, i.e., the differential diagnosis of IPD from APS. Utilizing unsupervised dimension reduction method and hierarchical clustering, Suh et al*.* divided FP-CIT PET scans into multiple groups and then evaluated the correlation between certain clusters and specific Parkinson symptoms, which depicted the heterogenous dopaminergic neurodegeneration patterns in parkinsonian [19]. However, it cannot directly characterize the probabilities of each parkinsonian syndrome and provide a diagnosis prediction when given an unseen scan.

In this study, we leveraged deep learning to extract informative imaging signatures from [^11^C]CFT PET scans to support the differential diagnosis of parkinsonian syndromes. A 3D deep residual convolutional neural network (termed as DAT-Net) was proposed, which can get access to the entire DAT image and involve the uptake distribution, content and context information among different regions. A large multi-cohort dataset of DAT imaging was collected to develop the DAT-Net and then to evaluate its performance. Furthermore, we investigated the decision mechanism of the deep learning network based on the state-of-the-art full-gradient saliency map method [20], which provides a view to understand the deep neural network and reveal the functional abnormal regions of patients with different syndromes indicated on the [^11^C]CFT PET scans.

## Material and Method

### Study Protocol

**Fig. 1:**
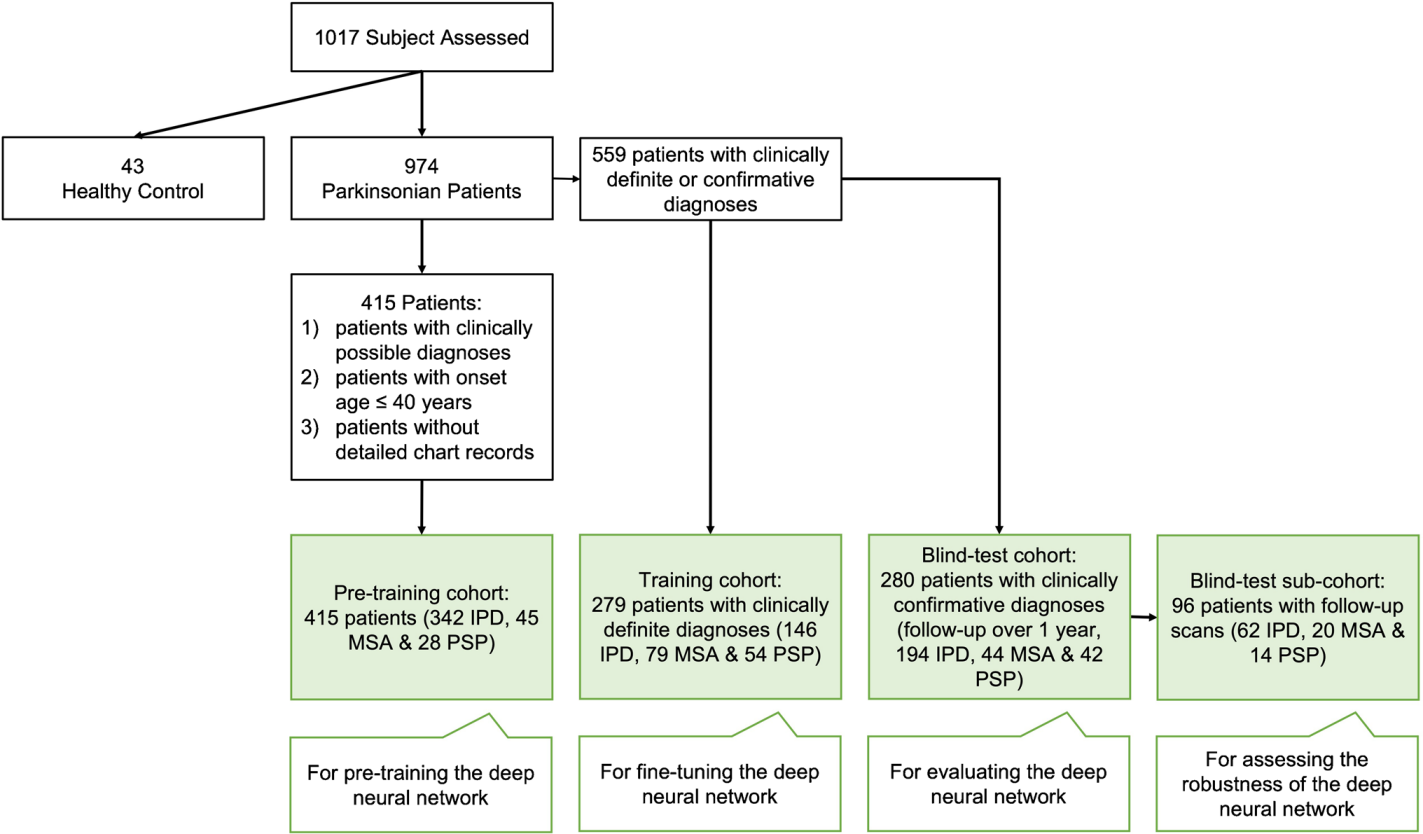
Study profile (the demographic and clinical data of included parkinsonian patients (*N* = 974) is given in Supplementary Table 1). IPD: idiopathic Parkinson's disease, MSA: multiple system atrophy, PSP: progressive supranuclear palsy, clinically definite diagnoses: diagnoses by the clinical experts after return visit but without a formal clinical follow-up, clinically confirmative diagnoses: diagnoses resulting from at least one formal clinical follow-up over two years after PET imaging

The study profile and detailed information of involved subjects are given in Fig. 1 and Supplementary Table 1. This study involved 1017 subjects including 43 healthy subjects as normal control (NC) and 974 parkinsonian (IPD, MSA or PSP) patients. All participants were enrolled from *Huashan Parkinsonian PET Imaging (HPPI) Database*, an oriental multimodal imaging database established to benchmark the imaging-based AI development for parkinsonism. The involved patients were routinely assessed by movement disorders specialists and underwent [^11^C]CFT PET imaging in Huashan Hospital (Shanghai, China). Before PET imaging, the routine MRI examination was performed for all the patients and those with structural brain abnormalities such as ischemia, white matter changes, mass lesions and hemorrhage was excluded from this study. The involved 43 healthy control subjects underwent the same clinical screening procedures, and the exclusion criteria included: (1) a history of neurological or psychiatric illness; (2) prior exposure to neuroleptic agents or drug addiction; (3) an abnormal neurological or MRI examination; (4) having used drugs with DAT blocking components. The clinical diagnoses of patients were made by the movement disorders specialists based on their return visits (at least once) after PET examination according to the latest clinical criteria [21–23]. All patients were divided into three cohorts termed as “clinically possible,” “clinically definite” and “clinically confirmative” diagnoses, where clinically definite diagnoses represent diagnoses by the clinical experts after return visit but without a formal clinical follow-up and clinically confirmative diagnoses represent diagnoses resulting from at least one formal clinical follow-up over two years after PET imaging. In this study, the pre-training cohort includes patients with a clinically possible diagnosis of IPD, MSA or PSP, the training cohort involves patients with a clinically definite diagnosis as well as NC, and the blind-test cohort is composed of the patients with a clinically confirmative diagnosis.

### Image Acquisition and Reconstruction

All patients stopped taking anti-parkinsonian medications (if used) at least 12 h before PET imaging and received an intravenous injection of the [^11^C]CFT (370 MBq). A 15-min three-dimensional emission scan was acquired one hour after the injection using the Biograph™ 64 HD PET/CT (Siemens Healthcare, Erlangen, Germany). The low-dose computed tomography was employed for the attenuation correction before the emission scan. PET images were then reconstructed using the three-dimensional ordered subset expectation maximization algorithm after the corrections for scatter, dead time and random coincidences. After that, the SPM5 software (Wellcome Department of Imaging Neuroscience, Institute of Neurology, London, UK), implemented in Matlab 7.4.0 (Mathworks Inc, Sherborn, MA), was utilized to spatially normalize these PET images into the Montreal Neurological Institute (MNI) brain space as previously described in [24, 25]. Individual MRI scans were not used for image preprocessing since these scans were performed in the routine clinical workup but not acquired according to a standardized protocol [25]. Finally, a three-dimensional Gaussian filter (10 mm full width at half maximum (FWHM)) was involved to smoothen the normalized PET images.

### Deep Neural Network and its Interpretation

**Fig. 2:**
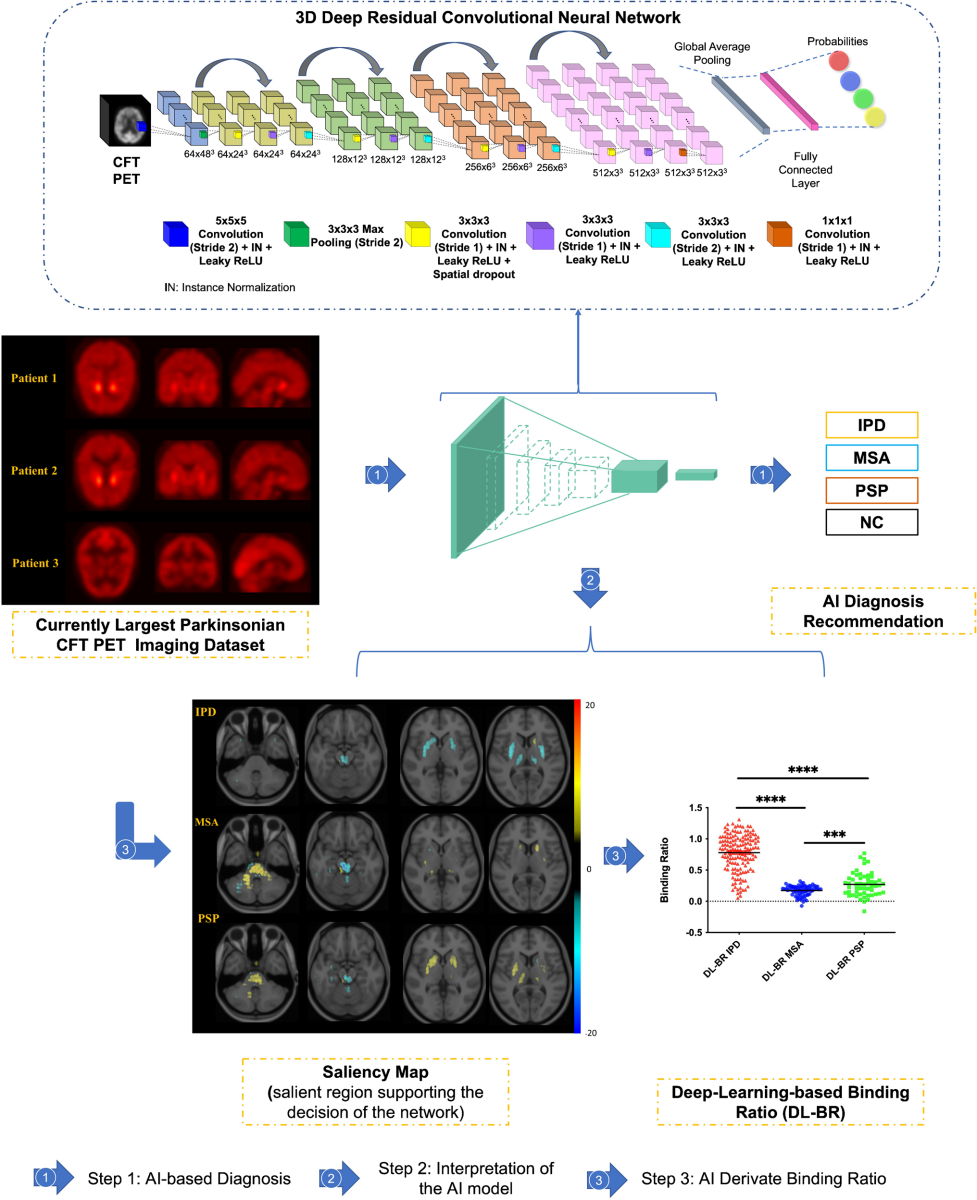
The schema of the developed DAT-Net for the differential diagnosis of parkinsonism. Step 1: the process of the AI-based diagnosis; Step 2: the interpretation of the derived AI Model via saliency map, where the assigned importance score to each voxel indicates its contribution in the decision making of the neural network. Step 3: the deep-learning-based binding ratio (DL-BR), where the mean counts within the most salient regions of the obtained deep neural network were regarded as the specific binding instead of using the putamen and caudate

The schema of the developed artificial intelligence (AI) method for the differential diagnosis of parkinsonism is illustrated in Fig. 2. Firstly, a DAT-Net was designed and trained for the AI-based diagnosis. Then, the saliency map was leveraged for the interpretation of the derived deep learning model, where the assigned importance score to each voxel indicates its contribution to the decision making of the neural network. Furthermore, a proposed deep-learning-based binding ratio (DL-BR) and deep-learning-guided radiomics (DL-radiomics) were analyzed for further understanding the mechanism of the AI-based method.

The architecture of the DAT-Net is shown in Fig. 2; it begins with a 5 × 5 × 5 convolution layer (stride 2) and a max pooling layer (stride 2) for down-sampling the input PET image. Then, there are four repeated encoder stacks with 3D convolutional layers (3 × 3 × 3) inside and a global average pooling layer to embed the input into a latent low-dimensional feature space. Finally, a fully connected layer and a softmax activation are utilized to conduct the diagnosis based on the learnt features. The skip-connection strategy was utilized in the designed network aiming at alleviating the vanishing gradient problem and simplifying the optimization [26]. The network was trained with the Adam optimizer with the learning rate of 10^–4^ under the supervision of the categorical cross-entropy loss. The early-stop strategy was leveraged with patience of 20 to prevent over-fitting. The pre-training cohort was firstly used to train the DAT-Net preliminarily, and the trained model was then fine-tuned on the training cohort. The performance of the developed model was evaluated with cross-validation (six-fold) on the training cohort and then independently tested on the blind-test cohort.

To make the DAT-Net explainable, we utilized full-gradient method [20] to calculate the saliency maps for assisting the interpretation of the implicit decision-making mechanism of the network. The full-gradient saliency map has the advantage of compressively assigning importance scores to both the input features and individual neurons in a network. The saliency scores in the map indicate the contribution of groups of pixels to the prediction results. (More methodology details such as the detailed network architecture are given in the supplementary material.)

### Binding Ratio Analysis

The binding ratio (BR) was utilized as a conventional method to analyze patients’ DAT images which is defined as $BR=({C}_{specific}-{C}_{nonspecific})/{C}_{nonspecific}$, where $C$ denotes the PET counts. The mean counts of occipital cortex were employed as the non-specific binding ${C}_{nonspecific}$, and the mean counts of bilateral putamen and caudate were regarded as the specific binding ${C}_{specific}$[25]. We also studied the accuracy of the binding ratio with referring to the mean counts of the most salient regions in saliency maps of the obtained deep neural network as the ${C}_{specific}$ (denoted as DL-BR). We utilized the top 70% salient regions to prevent the influence of the possible noise. (Detailed information can be found in supplementary material.)

### Radiomics Features Analysis

The radiomics features were extracted with the pyradiomics library [27] (version v3.0.1) implemented with the python. Radiomics features such as the first-order statistics, 3D shape-based features and gray-level co-occurrence matrix were extracted from the putamen and caudate regions for analysis. We further evaluated radiomics features extracted from the most salient regions of the obtained deep neural network, which was denoted as the deep-learning-guided radiomics (DL-radiomics) features.

### Combination of DAT imaging scans with demographic and clinical features

Demographic profiles (age, gender, symptom duration) and clinical assessment data (unified Parkinson's disease rating scale (UPDRS), Hoehn and Yahr stage) were collected, where the latter were acquired under an antiparkinsonian medication withdrawal > 12 h (if used). We applied the Extreme Gradient Boosting (XGBoost) [28], which is a decision-tree-based ensemble machine learning algorithm, to evaluate the performance of integrating information of both DAT scans and demographic/clinical features (multiple modalities) for the diagnosis of parkinsonism. The obtained DAT-Net-derived signatures (predicted possibilities of IPD, MSA, PSP) and demographic information/clinical data were jointly employed by the XGBoost to perform the multi-modality diagnosis. The XGBoost was trained on the training cohort, and the multi-modality diagnostic performance was then evaluated on the blind-test cohort.

### Statistics Analysis

This work calculated the area under the curve (AUC) and plotted the receiver operating characteristic (ROC) curves by the scikit-learn library in python. The Youden’s index was applied to estimate the optimal cutoff points of the ROC curves. The DeLong’s method (1988) was utilized to calculate the confidence intervals (CIs), and the confidence level of CIs in this work was 95%. The Chi-square test was employed to evaluate the performance difference, the Mann–Whitney U test was used to compare the radiomics features between two groups, and a two-sided p-value of less than 0.05 was considered significant. The sensitivity, specificity, positive predictive value (PPV) and negative predictive value (NPV) were employed to demonstrate the diagnostic performance of the DAT-Net.

## Results

### Performance of the DAT-Net in cross-validation

**Fig. 3:**
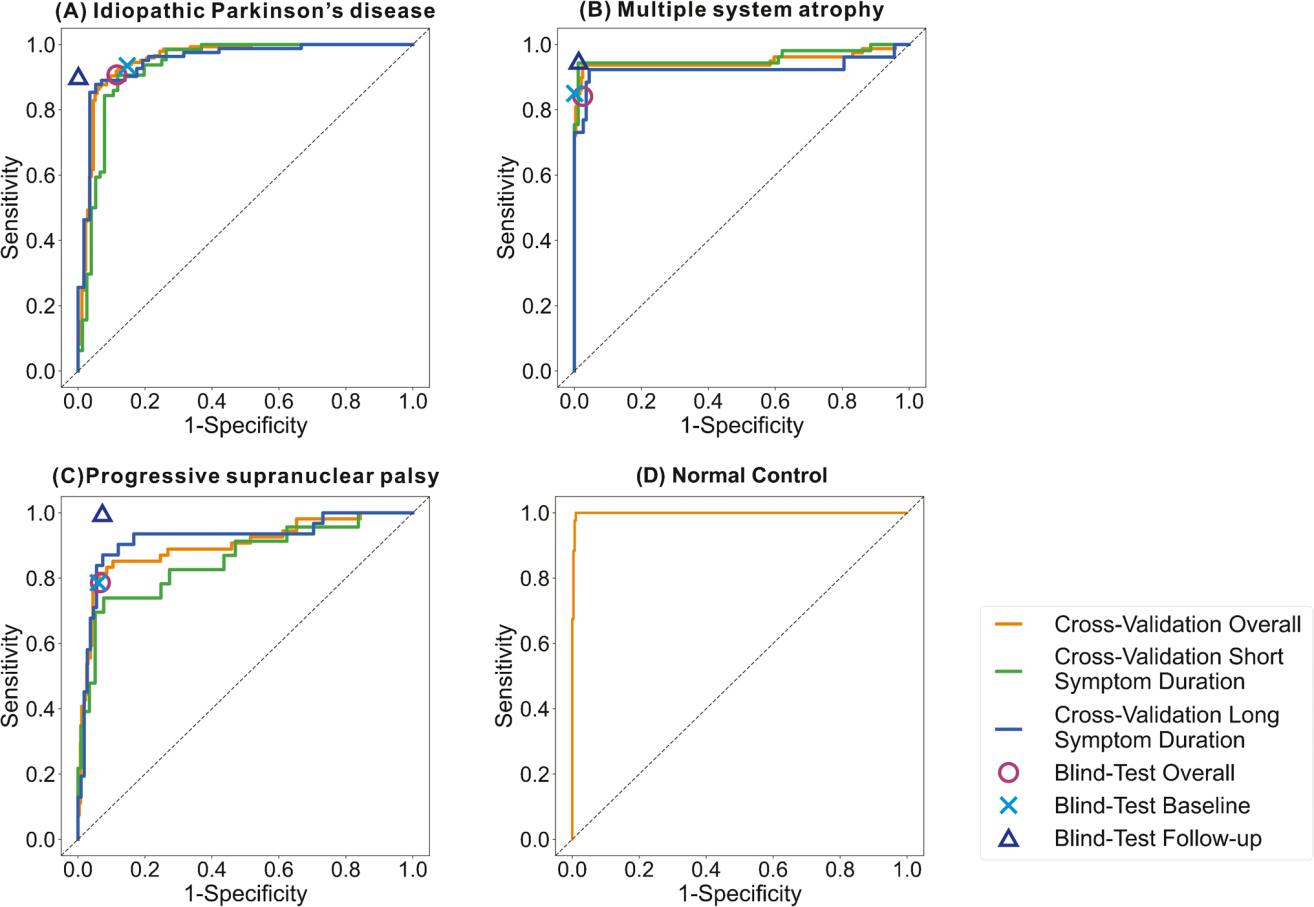
The performance of the proposed DAT-Net in the cross-validation and blind test (more details are given in Supplementary Table 2 and Table 3). In the cross-validation, short symptom duration represents patients with symptom duration ≤ 2 years and long symptom duration means patients with symptom duration > 2 years. In the blind-test phase, overall represents the results of all the tested 280 patients, baseline and follow-up denote the results of 96 patients that have both initial and repeated scans

The proposed DAT-Net obtained over or equal to the AUC of 0.900 for differential diagnosis of IPD, MSA, PSP and NC in the cross-validation phase (Fig. 3 and Supplementary Table 2). The obtained AUCs on the patients with long symptom durations (> 2 years) were higher than those with short symptom durations (≤ 2 years) for IPD and PSP, while for MSA, the DAT-Net achieved better AUC on patients with short symptom durations. The overall sensitivity of the differential diagnosis of IPD, MSA and PSP ranged from 81.5% to 93.7%, while the specificity ranged from 93.2% to 97.5% (Supplementary Table 2 overall). Generally, the performance of DAT-Net between patients with long symptom duration and patients with short symptom duration was comparable (*P* = 0.75 Chi-square test). When evaluating the performance of distinguishing parkinsonian patients with IPD or APS from NC, we found that the DAT-Net was a strong predictor with AUC of 0.998, the sensitivity of 100.0%, specificity of 98.9%, PPV of 93.5% and NPV of 100.0% (Supplementary Table 2).

### Performance of the DAT-Net in the blind test

**Fig. 4:**
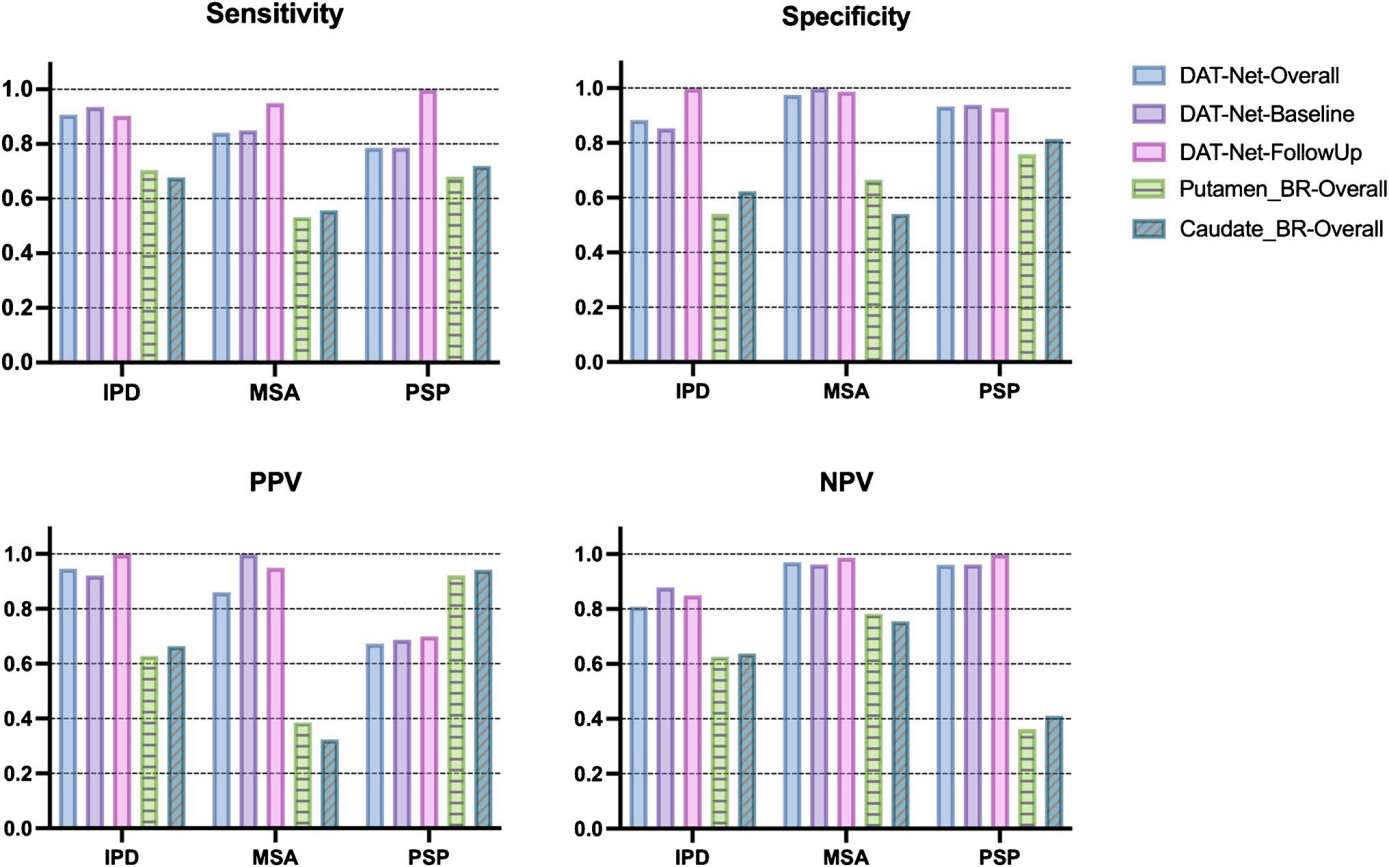
The performance of the DAT-Net for the differential diagnosis of the parkinsonian disorders evaluated on the blind-test cohort. The performance of the traditional BR quantification is also illustrated for comparison. The DAT-Net significantly outperformed BR quantification (*P* < 0.0001, Chi-square test). The detailed numbers are given in Supplementary Table 3

The effectiveness of the DAT-Net was further evaluated on the blind-test cohort. As demonstrated in Fig. 4, the deep neural network resulted in a sensitivity of 90.7% and specificity of 88.4% for differential diagnosis of IPD, with 84.1% and 97.5% for MSA as well as 78.6% and 93.3% for PSP, respectively (Supplementary Table 3). The performance was significantly higher than transitional BR quantification (*P* < 0.0001 Chi-square test, Fig. 4). The obtained overall performance in the blind test was comparable to cross-validation (*P* = 0.60 Chi-square test).

For the 96 patients in the blind test with both initial and repeated scans, we also evaluated the performance of the network referring to the initial (baseline) and repeated (follow-up) scans separately (Fig. 4 and Supplementary Table 3). Comparing the performance of follow-up with baseline, the overall diagnosis accuracy at follow-up is slightly superior with 92.7% vs 89.6% (*P* = 0.45, Chi-square test).

### Interpretation of the DAT-Net

**Fig. 5:**
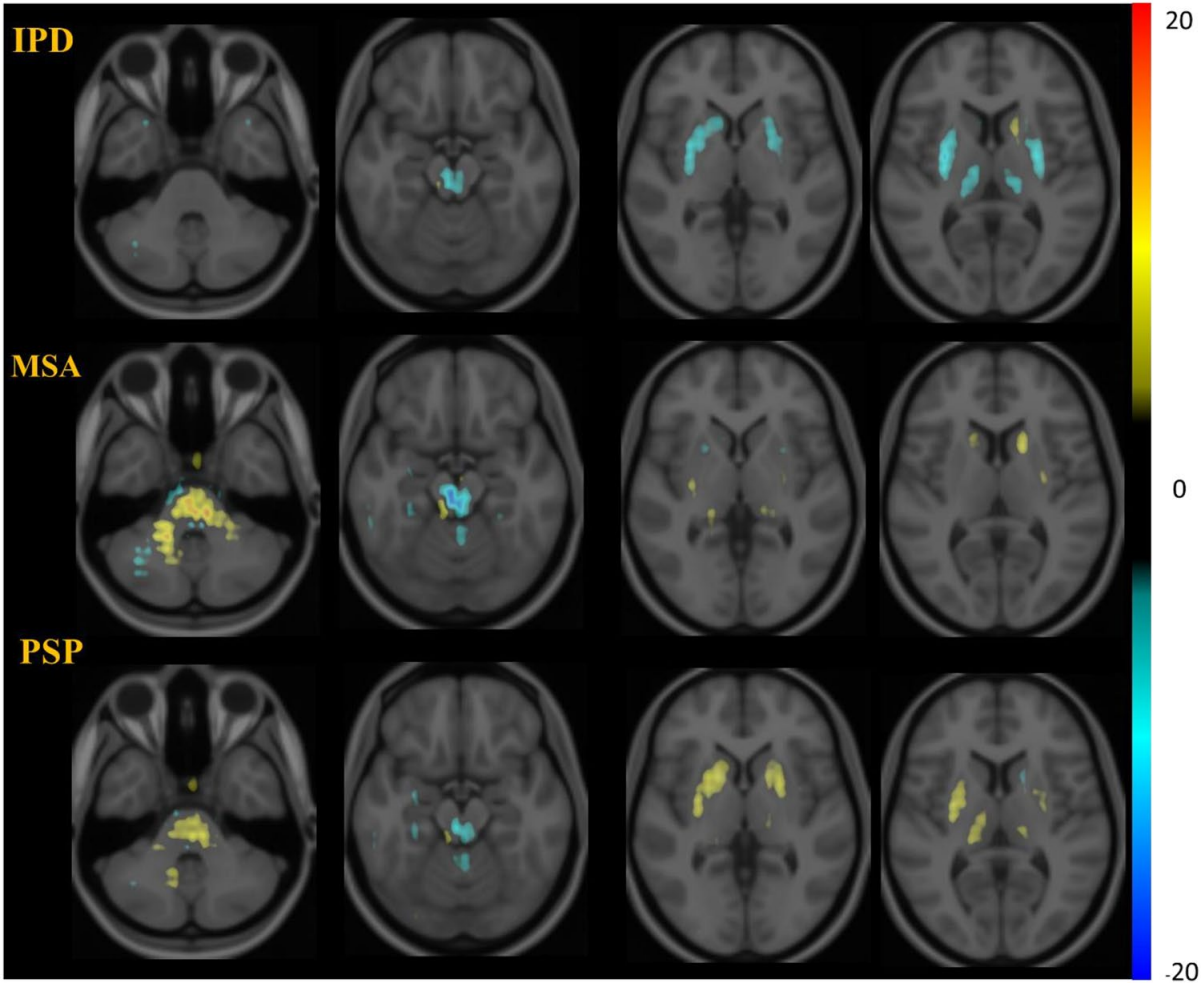
Visualization of average saliency maps of patients with idiopathic Parkinson’s disease (IPD), multiple system atrophy (MSA), progressive supranuclear palsy (PSP). These maps illustrate the characteristic regions supporting the determination of the DAT-Net. The color corresponds to the importance score indicating the contribution of a region

The saliency map, for the interpretation of deep neural networks, assigns an importance score to each voxel in the input image to indicate its contribution to the decision making of the neural network. We demonstrated the average saliency maps of patients with IPD, MSA or PSP in the training cohort (fused with template MRI). In general, the saliency maps illustrated that the most contributed regions determining the diagnosis of IPD, MSA and PSP are highly related to several parkinsonism-related regions such as putamen, caudate and midbrain (Fig. 5).

### Deep-learning-guided Binding Ratio Analysis

**Fig. 6:**
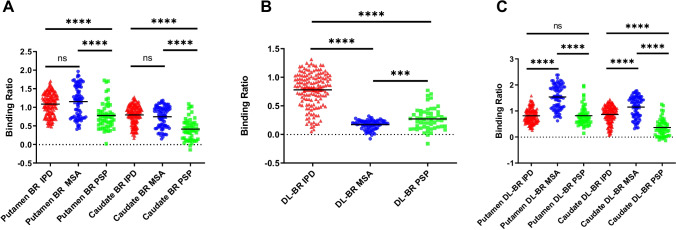
Conventional and deep-learning-based binding ratio analysis of the DAT images. (**A**) Conventional putamen and caudate binding ratio. (**B**) Deep-learning-based binding ratio (DL-BR), where the mean counts of the most salient regions in saliency maps of the obtained deep neural network as the specific binding instead of using the putamen and caudate. (**C**) Region-specific DL-BR, where the most saliency regions (referring obtained saliency maps) located in the putamen and caudate were leveraged to calculate the specific binding

The conventional and deep-learning-based binding ratios of the DAT images are analyzed in this section. Figure 6(A)  illustrates the conventional putamen and caudate binding ratios (BRs). Figure 6(B) demonstrates deep-learning-based binding ratio (DL-BR), where the mean counts within the most salient regions of the obtained deep neural network were regarded as the specific binding instead of using the putamen and caudate. Figure 6(C) shows the region-specific DL-BR, where the most saliency regions of the network located in the putamen and caudate were leveraged as the reference regions to calculate the specific binding. Figure 6(A)  indicates that there was no significant difference of putamen and caudate BRs between IPD and MSA groups (putamen: *P* = 0.24, caudate: *P* = 0.30), but significant differences could be found between both IPD vs PSP and MSA vs PSP groups (*P* < 0.0001), respectively. However, the DL-BRs demonstrated significant differences among the three comparison groups (IPD vs MSA: *P* < 0.0001, IPD vs PSP: *P* < 0.0001 and MSA vs PSP: *P* < 0.001 (Fig. 6(B)). For region-specific DL-BRs, we found the same conclusion as DL-BRs except that there was no significant difference between the IPD group and PSP group in putamen DL-BR (*P* = 0.42, Fig. 6(C)).

### Deep-learning-guided Radiomics Analysis

**Fig. 7:**
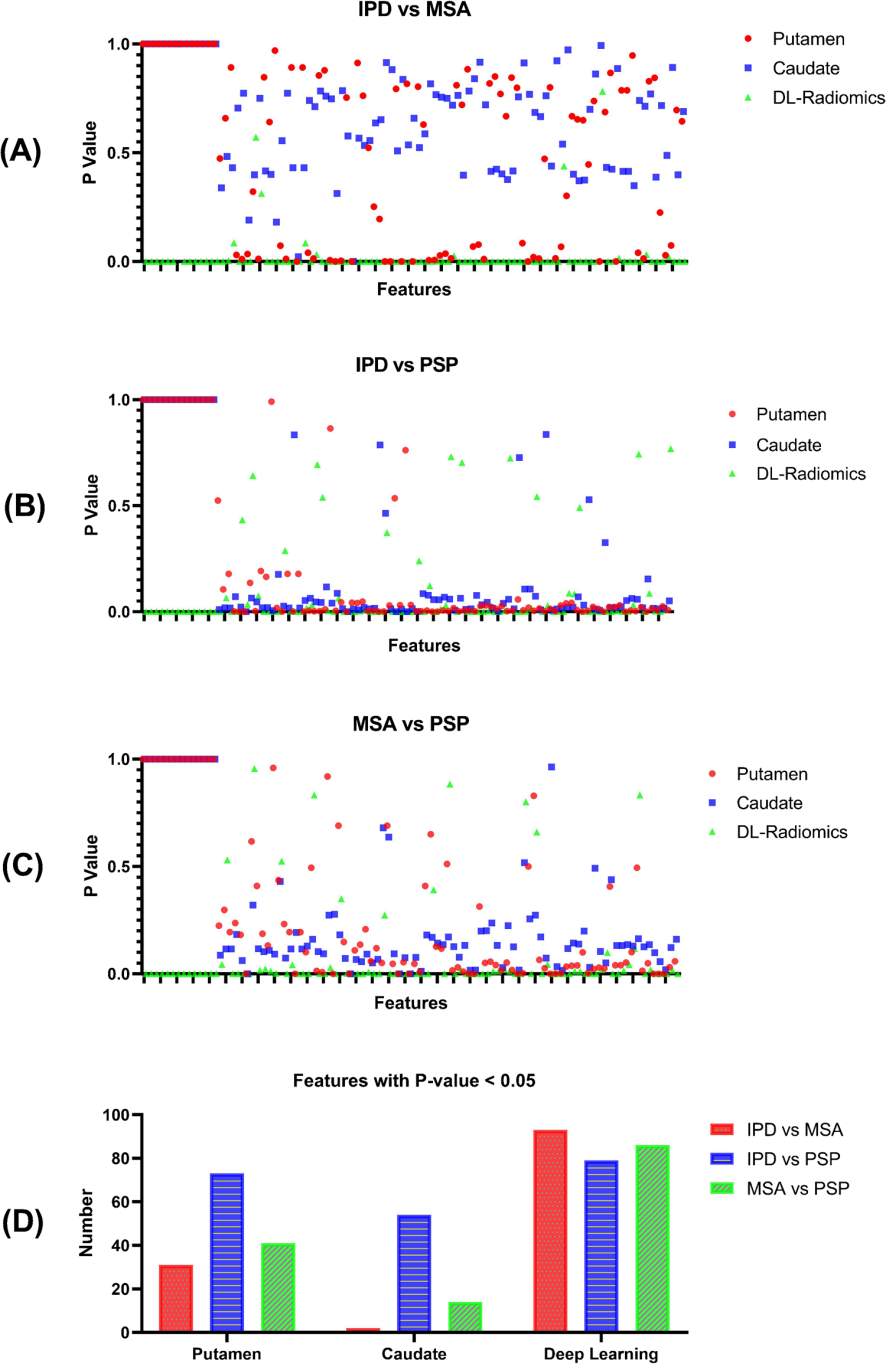
Comparison between deep-learning-guided radiomics (DL-radiomics) and conventional radiomics (from putamen and caudate regions)**.** The Mann–Whitney U test was used to compare the radiomics features between two groups, and a two-sided p value of less than 0.05 was considered significant

We further evaluated the deep-learning-guided radiomics features, i.e., radiomics features extracted from the most salient regions of the obtained deep neural network and then compared them with the conventional radiomics features from the putamen and caudate regions. The statistical difference of deep-learning-guided and conventional radiomics features between different groups (IPD vs MSA, IPD vs PSP and MSA vs PSP) is shown in Fig. 7(A-C), and it can be found that there were average above 78.1% more deep-learning-guided radiomics features with significant differences among IPD, MSA and PSP compared to conventional radiomics features (Fig. 7(D)). To be specific, there existed 93, 79 and 86 DL-radiomic features that have significant differences (*P *< 0.05) between IPD and MSA, IPD and PSP, as well as MSA and PSP, respectively (100 features in total), while the number of conventional putamen radiomics features with significant difference between groups was 31, 73, and 41 and the number regarding conventional caudate radiomics features was 2, 54 and 14.

### Combining the DAT imaging scans with demographic and clinical features

**Fig. 8:**
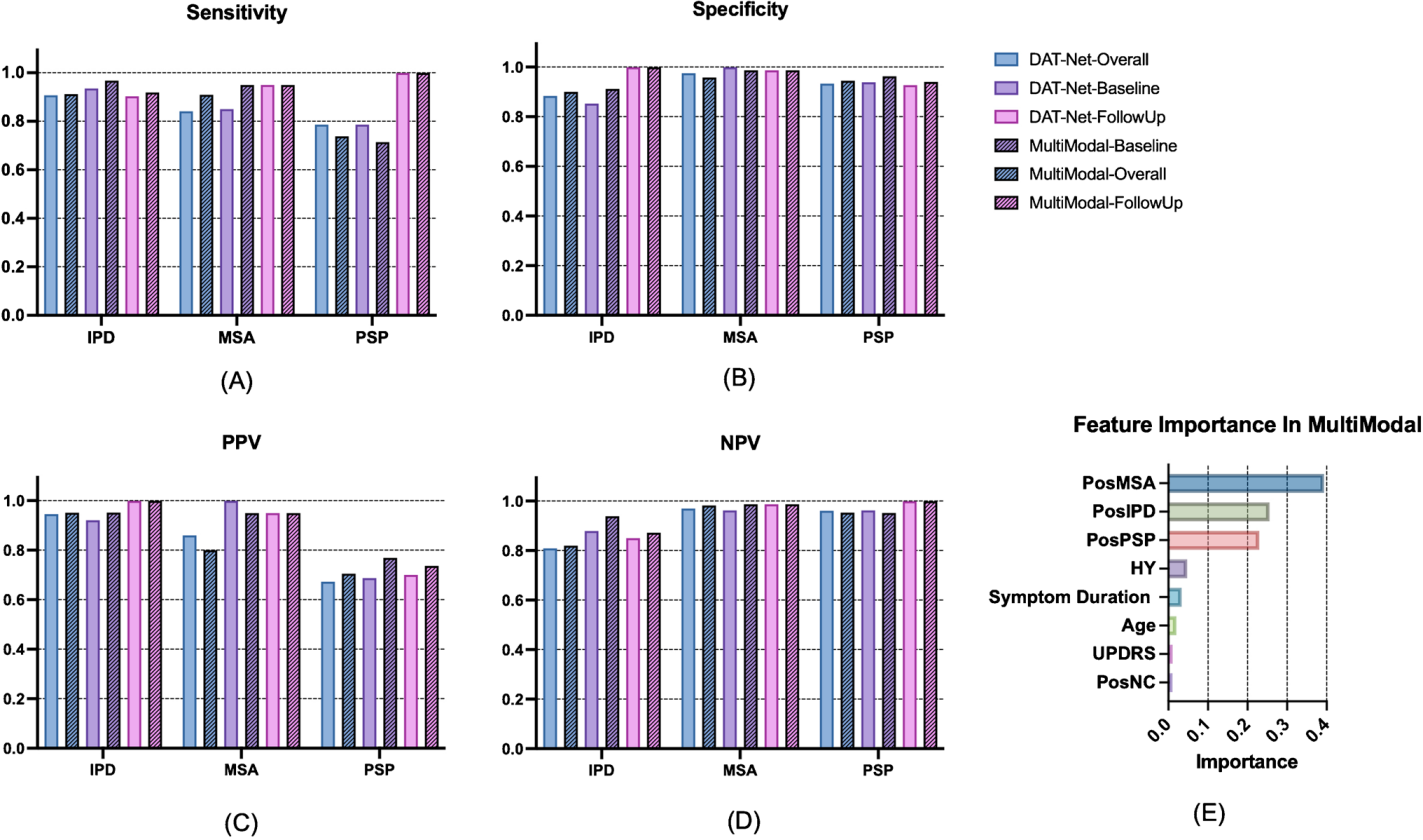
Multi-modality study: the performance and feature importance when combining the DAT imaging scans with demographic and clinical features for the differential diagnosis of parkinsonism based on the XGBoost classifier. Four DAT imaging-derived features, i.e., prediction possibilities of IPD, MSA, PSP and NC obtained by the DAT-Net together with demographic and clinical features including age, gender, symptom duration, UPDRS, Hoehn and Yahr stage were involved into the model. The detailed numbers are given Supplementary Table 4

We evaluated the performance of combining the DAT imaging scans and demographic and clinical features (multi-modality) compared to utilizing DAT imaging only (single-modality). Generally, the multi-modality procedure slightly outperformed the single-modality procedure (overall accuracy 89.3% vs 87.9%, *P* = 0.60). Specifically, the results (sensitivity, specificity, PPV, NPV) after leveraging multi-modality data were better for the differential diagnosis of IPD on overall, baseline subgroup and follow-up subgroup of patients in blind-test cohort. And the performance of multi-modality was equal or superior to that of single DAT imaging modality for differentiating IPD, MSA and PSP evaluated on follow-up scans (Fig. 8, Supplementary Table 4).

In our multi-modality method, we included DAT imaging derived feature, i.e., the prediction possibilities of IPD, MSA, PSP, NC obtained by the DAT-Net, as well as demographic and clinical features including age, gender, symptom duration, UPDRS, Hoehn and Yahr stage. We then evaluated the contribution of the above-mentioned nine features, and the results are shown in Fig. 8(E). The most important five features were possibilities of MSA, IPD and PSP, Hoehn and Yahr stage, as well as symptom duration, which indicates that DAT-imaging-derived features contribute the main part in the decision of multi-modality classifier. Besides, the gender feature showed no contribution. (Feature importance is equal to zero.)

## Discussion

Evaluated on one of the largest available datasets with 1017 subjects, our preliminary results demonstrated that the potential of the [^11^C]CFT PET scans for differentiating IPD and APS subgroups based on the proposed DAT-Net, which might benefit from deep learning’s ability for decoding critical information from DAT imaging. In current clinical practice, DAT imaging is considered unsuitable for the reliable differentiation of IPD and APS subtypes such as MSA and PSP[29, 30]. While the conventional differential diagnosis referring to DAT imaging is based on quantitative analysis such as the DAT binding ratio (BR) quantification [4–6] in specific regions such as putamen and caudate, the numerous information of DAT imaging was neglected. In the current study, taking advantage of deep-learning method, we were able to dig deeper into this classical functional imaging modality and successfully expanded its significance for disease diagnosis with DAT-Net, DL-BR and DL-radiomics.

The performance of the DAT-Net was evaluated both in the cross-validation stage and blind-test stages. The success of the neural network mainly benefited from its capacity to access the global information of entire PET scans and analyze multiple regions as well as their correlation simultaneously, which was different from the traditional method that only focused on certain slices and certain regions. The relatively comparable performance in these two stages showed the robustness of our proposed network between different cohorts. The high accuracy on the patients with short symptom duration (Fig. 3, Supplementary Table 2) and patients at baseline (Fig. 4, Supplementary Table 3) suggested the potential of DAT-Net for early diagnosis. Patients with longer symptom durations were supposed to have more extensive changes in the brain as disease progression. Our network obtained comparable performance between patients with short symptom duration/at baseline and with long symptom duration/at follow-up, which also implied that the proposed network was sensitive to brain changes on the DAT imaging scans, i.e., even slight changes in the early stage can be recognized by the DAT-Net to provide a similar accurate diagnosis, compared to that made after referring to more significant changes at follow-up (longer symptom duration). Besides, our proposed network achieved remarkable performance in the differential diagnosis of parkinsonian patients (with IPD or APS) from NC, which confirmed the ability of DAT imaging for the diagnosis of parkinsonian patients from NC due to the significant striatal DAT loss in the image.

The saliency maps suggested that the network paid attention mainly to putamen, caudate and midbrain, which meant that these regions were assigned higher importance scores and therefore contributed mainly to the final prediction of the network, though the remaining regions also showed contribution. The putamen and caudate, which were regarded as the leading corresponding brain regions for disease progression [31, 32], were the most common included regions in the traditional conventional quantitative analysis [33, 34]. For the midbrain, as a vital structure in dopamine signaling, the heterogeneity of dopamine neurons was considered to underpin the variety of clinical symptoms [35]. Moreover, [^11^C]CFT also displays a high affinity for serotonin transporters (SERT) in addition to DAT in the midbrain and previous studies have shown that midbrain SERT distribution is significantly different between PD and MSA-P groups or between PD and PSP groups [34, 36], which may be another important factor suggesting the DAT-Net to pay attention to the tracer binding in midbrain for the differential diagnosis. All these previous findings supported that the detected regions in the saliency maps were in accordance with the key structures of the underlying pathological mechanisms.

Comparing the performance of the conventional BR and our designed DL-BR among IPD, MSA and PSP groups, we found the conventional putamen and caudate BR had limited potential for differential diagnosis, which was consistent with existing research results [29, 30]. However, there were significant differences among IPD, MSA and PSP groups if evaluating with the new designed DL-BR. This result was in line with our previous DAT-Net analysis that the neural network can access the entire PET scan and then learn the specific regions with distinguishable PET information for the differential diagnosis of IPD, MSA and PSP. Similarly, compared to conventional radiomics features, more DL-radiomics features showed significant differences between different groups (IPD vs MSA, IPD vs PSP and MSA vs PSP), which also indicated the advantage of the learned specific regions by the DAT-Net. Subregional patterns analysis of dopamine transporter loss was suggested as a potential way to improve the differential diagnosis of parkinsonism [37]. The improved performance of the subregions defined by deep learning in this study may provide a complementary tool to identify more efficient subregional patterns. Furthermore, the neural network can not only locate the diagnosis-informative regions but also assign weights on each voxel within these regions. Therefore, it may be more supportive than DL-BR and DL-radiomics in the differential diagnosis. To be specific, compared to conventional quantitative analysis which assigned the same weight on the uptake of each voxel when analyzing certain regions, the neural network allowed assigning different automatically learnt optimal weights on different voxels (Fig. 5). These weights may reflect the inter-correlation among regions, which may be dependent on the pathogenesis of different types of parkinsonism. While all suffered from dopaminergic dysfunction, previous studies showed that IPD, MSA and PSP had different preferential subregional decreases in striatal DAT binding[37] and different speeds of dopaminergic degeneration [38]. Considering the different directions and different speeds during the progression of dopamine transporter loss, future investigation of these interrelations may assist the understanding of the pathway behind the disease. Overall, the combination of the diagnosis-informative sub-region and the interrelation-determined weights has improved the ability of the network in the differential diagnosis of IPD, MSA and PSP.

Unsurprisingly, our experiments also illustrated that leveraging multi-modality data slightly outperformed only utilizing image modality, which might be due to that the image-only modality itself already achieved a relative high accuracy for differentiation. While the most important three features were all extracted from the DAT imaging scans, we inferred that the image modality accounted for the main contribution of the proposed multi-modality method and the demographic and clinical features were beneficial for improving the diagnosis performance and robustness.

As a data-driven method, data played a central role in the development of neural networks. Sufficient training data were helpful for the network to learn features of diversity cases and therefore could improve the performance of the network and prevent them from over-fitting. Although the relatively large dataset in this study allowed for a comprehensive understanding of this neural network within IPD and APS, the influence of the physical complexity of imaging data on such models remained to be further explored. And therefore, we would like to evaluate the performance of our DAT-Net when addressing scans obtained from different devices. Another limitation of the present study was that the entire experiment was based on a retrospective cohort and multi-center prospective studies were still needed to further confirm the protentional of this method in the differential diagnosis of parkinsonism based on DAT imaging. Furthermore, we utilized one possible multi-modality fusion method to synthesize multimodal information in this work. In the future, other fusion methods such as gating-based attention-based late fusion [39] will be leveraged to further improve the multimodal diagnosis performance. Another interesting future work is to evaluate the potential of utilizing the pre-trained DAT-Net (trained on [^11^C]CFT PET) for the differential diagnosis of parkinsonism on FP-CIT SPECT. There exist large gaps of tracer, modality and ethnics between the two modalities. The domain-adversarial training strategy [40] may have the potential to alleviate the influence of the modality gap and further extend the proposed method of this study.

## Conclusion

In this paper, we developed a 3D deep residual convolutional neural network for the automated differential diagnosis of parkinsonism based on the [^11^C]CFT PET scans. The experiment results in the cross-validation phase and the blind test phase demonstrated the relatively high accuracy and the generalization ability of the proposed method. The saliency maps of the deep neural network, which indicated the functional basis and distinguishable regions recognized by the network, were consistent with known neuropathological processes in parkinsonism. These findings suggest that employing the deep neural network in the analysis of DAT scans has the potential to assistant neurologists in making reasonable diagnoses of the parkinsonian syndromes at an early stage.

## Supplementary Information

Below is the link to the electronic supplementary material.

Supplementary file1 (DOCX 85 KB)

## Data Availability

All data included in this study will be available to the scientific community upon completion of the non-disclosure agreement (NDA) with the corresponding author according to international data protection regulations.

## Code availability

Our code is available for download at: https://github.com/Louis-YuZhao/DAT-NET.git.
