# Supplementary material for: Decoding the dopamine transporter imaging for the differential diagnosis of parkinsonism using deep learning
Source: Eur J Nucl Med Mol Imaging. 2022 May 19;49(8):2798–811. doi: 10.1007/s00259-022-05804-x (PMC9206631; doi:10.1007/s00259-022-05804-x)
Supplement: Supplementary file 1 — Supplementary file1 (DOCX 85 KB) [file 259_2022_5804_MOESM1_ESM.docx]

**Supplementary**

**Abstract**

**Purpose:** This work attempts to decode the discriminative information in dopamine transporter (DAT) imaging using deep learning for the differential diagnosis of parkinsonism.

**Methods:** This study involved 1017 subjects who underwent DAT PET imaging ([^11^C]CFT) including 43 healthy subjects and 974 parkinsonian patients with idiopathic Parkinson’s disease (IPD), multiple system atrophy (MSA) or progressive supranuclear palsy (PSP). We developed a 3D deep convolutional neural network to learn distinguishable DAT features for the differential diagnosis of parkinsonism. A full-gradient saliency map approach was employed to investigate the functional basis related to the decision mechanism of the network. Furthermore, deep-learning-guided radiomics features and quantitative analysis were compared with their conventional counterparts to further interpret the performance of deep learning.

**Results:** The proposed network achieved area under the curve of 0.953 (sensitivity: 87.7%, specificity: 93.2%), 0.948 (sensitivity: 93.7%, specificity: 97.5%), and 0.900 (sensitivity: 81.5%, specificity: 93.7%) in the cross-validation, together with sensitivity of 90.7%, 84.1%, 78.6% and specificity of 88.4%, 97.5% 93.3% in the blind test for the differential diagnosis of IPD, MSA and PSP, respectively. The saliency map demonstrated the most contributed areas determining the diagnosis located at parkinsonism-related regions, e.g., putamen, caudate and midbrain. The deep-learning-guided binding ratios showed significant differences among IPD, MSA and PSP groups (P<0.001), while the conventional putamen and caudate binding ratios had no significant difference between IPD and MSA (P=0.24 and P=0.30). Furthermore, compared to conventional radiomics features, there exist average above 78.1% more deep-learning-guided radiomics features had significant differences among IPD, MSA and PSP.

**Conclusion:** This study suggested the developed deep neural network can decode in-depth information from DAT and showed potential to assist the differential diagnosis of parkinsonism. The functional regions supporting the diagnosis decision were generally consistent with known parkinsonian pathology but provided more specific guidance for feature selection and quantitative analysis.

**Supplementary Table 1** The demographic and clinical data of included parkinsonian patients (N=974).

|  |  | Pre-training Cohort^1^ | Training Cohort^2^ | | | Blind-test Cohort^3^ | | |
| --- | --- | --- | --- | --- | --- | --- | --- | --- |
|  |  |  | Overall | Short Symptom Duration | Long Symptom Duration | Overall | Baseline | Follow-up |
| Idiopathic Parkinson Disease* | Number of patients | 342 | 146 | 64 | 82 | 194 | 62 | 62 |
|  | Sex (male/female) | 202/140 | 86/60 | 35/29 | 51/31 | 123 / 71 | 44 / 18 | 44 / 18 |
|  | Age at CFT PET (years) | 53.6±14.7 | 60.4±9.6 | 59.4±9.4 | 61.2±9.7 | 58.5 ± 8.7 | 59.7 ± 7.9 | 62.6 ± 8.0 |
|  | Symptom duration at CFT PET (months) | 40.8±43.4 | 39.3±42.6 | 13.1±5.4 | 59.6±47.7 | 40.3 ± 39.8 | 46.5 ± 46.1 | 81.7 ± 39.6 |
|  | Hoehn and Yahr stage | N/A | 2.0±0.9 | 1.7±0.7 | 2.3±1.0 | 1.9 ± 0.9 | 1.9 ± 1.0 | 2.2 ± 0.7 |
|  | UPDRS | N/A | 23.6±12.5 | 17.9±8.5 | 28.0±13.4 | 22.9 ± 12.7 | 24.0 ± 14.6 | 26.9 ± 11.8 |
|  | Clinical follow-up (months) | N/A | N/A | N/A | N/A | 52.0 ± 27.9 | 58.2 ± 29.1 | N/A |
| Multiple System Atrophy | Number of patients (MSA-C/MSA-P) | 45 | 79 (33/46) | 53 (23/30) | 26 (10/16) | 44 | 20 | 20 |
|  | Sex (male/female) | 32/13 | 39/40 | 25/28 | 14/12 | 29 /15 | 14 / 6 | 14 / 6 |
|  | Age at CFT PET (years) | 59.6±11.7 | 58.2±8.8 | 57.3±9.1 | 60.0±8.1 | 57.4 ± 7.3 | 58.15 ± 8.6 | 60.5 ± 8.5 |
|  | Symptom duration at CFT PET (months) | 26.0±17.9 | 22.8±16.8 | 14.2±5.9 | 40.5±18.0 | 28.7 ± 16.1 | 27.0 ± 14.1 | 54.0 ± 14.1 |
|  | Hoehn and Yahr stage | N/A | 3.1±0.9 | 2.9±0.8 | 3.5±0.9 | 2.8 ± 0.7 | 2.8 ± 0.5 | 3.6 ± 0.9 |
|  | UPDRS (rated in OFF) | N/A | 30.5±14.9 | 27.4±12.4 | 36.7±17.5 | 27.1 ± 12.7 | 24.0 ± 10.4 | 38.2 ± 12.1 |
|  | Clinical follow-up (months) | N/A | N/A | N/A | N/A | 34.8 ± 19.0 | 42.0 ± 16.5 | N/A |
| Progressive Supranuclear Palsy* | Number of patients | 28 | 54 | 23 | 31 | 42 | 14 | 14 |
|  | Sex (male/female) | 19/9 | 33/21 | 13/10 | 20/11 | 28 / 14 | 12 / 2 | 12 / 2 |
|  | Age at CFT PET (years) | 63.7±9.0 | 66.5±7.5 | 63.5±7.9 | 68.8±6.4 | 65.2 ± 6.9 | 65.0 ± 7.8 | 67.3 ± 7.6 |
|  | Symptom duration at CFT PET (months) | 32.4±21.4 | 36.0±23.4 | 15.7±5.7 | 50.9±19.9 | 37.0 ± 24.9 | 33.6 ± 24.4 | 60.5 ± 25.6 |
|  | Hoehn and Yahr stage | N/A | 3.4±0.8 | 3.0±0.7 | 3.6±0.8 | 3.0 ± 1.0 | 2.7 ± 1.1 | 3.6 ± 0.9 |
|  | UPDRS | N/A | 30.2±12.8 | 28.0±12.0 | 31.9±13.3 | 26.1 ± 11.8 | 22.4 ± 11.3 | 34.4 ± 17.3 |
|  | Clinical follow-up (months) | N/A | N/A | N/A | N/A | 27.8 ± 16.5 | 37.4 ± 12.6 | N/A |

^1^ The pre-training cohort includes 415 patients for the preliminary training of the deep neural network.

^2^ The training cohort includes 279 patents with clinically definite diagnosis according to latest diagnostic criteria for the fine-tuning of the pre-trained deep neural network and the evaluation during the development of the DAT-Net. Short symptom duration represents patients with symptom duration ≤ 2 years and long symptom duration means patients with symptom duration > 2 years.

^3^ The blind-test cohort includes 280 patients with clinically confirmative diagnosis after follow-up for independent and in-depth test of the developed DAT-Net. Among them, 96 patients had both initial PET scan (Baseline) and repeated PET scan (Follow-up).

Data are shown as mean ± standard deviation.

N/A: not available

**Supplementary Table 2** Performance of the DAT-Net for the differential diagnosis of the parkinsonian disorders evaluated on the training cohort. The evaluation is performed using cross-validation during the development of the DAT-Net.

|  |  | Overall | Overall Patients | Short Symptom Durations (≤ 2 years) | Long Symptom Durations (> 2 years) |
| --- | --- | --- | --- | --- | --- |
| Idiopathic Parkinson Disease (IPD) | ROCAUC | 0.953 (0.930-0.976) | 0.938 (0.909-0.969) | 0.931 (0.887-0.975) | 0.950 (0.912-0.988) |
|  | Sensitivity | 87.7% (81.2%-92.5%) | 86.3% (79.6%-91.4%) | 90.6% (80.7%-96.5%) | 87.8% (78.7%-94.0%) |
|  | Specificity | 93.2% (88.4%-96.4%) | 92.5% (86.6%-96.3%) | 88.2% (78.7%-94.4%) | 94.7% (85.4%-98.9%) |
|  | PPV | 91.4% (85.6%-94.9%) | 92.6% (86.9%-95.5%) | 86.6% (76.2%-94.8%) | 96.0% (88.6%-98.1%) |
|  | NPV | 90.1% (84.7%-94.7%) | 86.0% (79.2%-92.9%) | 91.8% (82.9%-96.2%) | 84.4% (73.5%-96.4%) |
| Multiple System Atrophy (MSA) | ROCAUC | 0.948 (0.907-0.991) | 0.947 (0.904-0.990) | 0.958 (0.913-1.000) | 0.925 (0.833-1.000) |
|  | Sensitivity | 93.7% (85.8%-97.9%) | 93.7% (85.8%-97.9%) | 94.3% (84.3%-98.8%) | 92.3% (74.9%-99.1%) |
|  | Specificity | 97.5% (94.7%-99.1%) | 97.0% (93.6%-98.9%) | 98.9% (93.8%-100.0%) | 95.6% (90.0%-98.5%) |
|  | PPV | 92.5% (84.8%-97.5%) | 92.5% (84.8%-97.5%) | 98.0% (89.7%-99.6%) | 82.8% (66.6%-97.7%) |
|  | NPV | 97.9% (95.1%-99.2%) | 97.5% (94.1%-99.1%) | 96.6% (90.3%-99.9%) | 98.2% (93.1%-99.4%) |
| Progressive Supranuclear Palsy (PSP) | ROCAUC | 0.900 (0.845-0.954) | 0.891 (0.834-0.948) | 0.855(0.757-0.953) | 0.921 (0.855-0.987) |
|  | Sensitivity | 81.5% (68.6%-90.7%) | 81.5% (68.6%-90.7%) | 73.9% (51.6%-89.8%) | 87.1% (70.2%-96.4%) |
|  | Specificity | 93.7% (90.0%-96.3%) | 92.4% (88.2%-95.5%) | 92.3% (85.9%-96.4%) | 92.6% (85.9%-96.7%) |
|  | PPV | 72.1% (61.3%-85.2%) | 72.1% (61.2%-85.2%) | 65.4% (49.0%-85.4%) | 77.1% (62.2%-93.0%) |
|  | NPV | 96.2% (92.6%-97.8%) | 95.4% (91.2%-97.3%) | 94.7% (87.1%-97.6%) | 96.2% (89.7%-98.3%) |
| Normal Control (NC) | ROCAUC | 0.998 (0.996-1.000) |  | / | / |
|  | Sensitivity | 100.0% (91.8%-100.0%) |  | / | / |
|  | Specificity | 98.9% (96.9%-99.8%) |  | / | / |
|  | PPV | 93.5% (82.9%-100.0%) |  | / | / |
|  | NPV | 100.0% (98.6%-100.0%) |  | / | / |

ROCAUC denotes the area under the receiver operating characteristic curve. PPV and NPV represent positive predictive value and negative predictive value.

Overall Patients：The performance of DAT-Net on all patients who were diagnosed with IPD, MSA or PSP (without considering NC cases). These patients can be divided into two subgroups according to the symptom duration. When calculating the AUCs of short or long symptom duration, we only consider all patients since those NC subjects have no short/long labels.

**Supplementary Table 3** Performance of the DAT-Net for the differential diagnosis of the parkinsonian disorders evaluated on the blind-test cohort.

|  |  | DAT-Net | | | Putamen_BR | Caudate_BR |
| --- | --- | --- | --- | --- | --- | --- |
|  |  | Overall^1^ | Baseline^2^ | Follow-up^3^ | Overall^1^ | Overall^1^ |
| Idiopathic Parkinson Disease | Sensitivity | 90.7% | 93.5% | 90.3% | 70.5% | 67.8% |
|  | Specificity | 88.4% | 85.3% | 99.9% | 54.1% | 62.4% |
|  | PPV | 94.6% | 92.1% | 99.9% | 62.8% | 66.4% |
|  | NPV | 80.9% | 87.9% | 85.0% | 62.6% | 63.8% |
| Multiple System Atrophy | Sensitivity | 84.1% | 85.0% | 95.0% | 53.2% | 55.7% |
|  | Specificity | 97.5% | 99.9% | 98.7% | 66.5% | 54.0% |
|  | PPV | 86.0% | 99.9% | 95.0% | 38.5% | 32.4% |
|  | NPV | 97.0% | 96.2% | 98.7% | 78.2% | 75.5% |
| Progressive Supranuclear Palsy | Sensitivity | 78.6% | 78.6% | 99.9% | 68.0% | 72.0% |
|  | Specificity | 93.3% | 93.9% | 92.7% | 75.9% | 81.5% |
|  | PPV | 67.3% | 68.7% | 70.0% | 92.2% | 94.2% |
|  | NPV | 96.1% | 96.2% | 99.9% | 36.3% | 41.1% |

^1^ The statistics of Overall summarizes the accuracy of all the 280 patients on initial [^11^C]CFT PET scans.

^2^ The statistics of Baseline summarizes the accuracy of 96 patients (with both initial and repeated PET scans) on the initial [^11^C]CFT PET scans.

^3^ The statistics of Follow-up summarizes the accuracy of 96 patients (with both initial and repeated PET scans) on the repeated [^11^C]CFT PET scans.

PPV and NPV represent positive predictive value and negative predictive value.

**Supplementary Table 4** Combining demographic and clinical features with [^11^C]CFT PET scans: Performance of the differentiation of the parkinsonian disorders with leveraging multi-modality data including [^11^C]CFT PET scans and clinical information (age, gender, symptom duration, UPDRS, Hoehn and Yahr stage) evaluated on the blind-test cohort.

|  |  | Overall^1^ | Baseline^2^ | Follow-up^3^ |
| --- | --- | --- | --- | --- |
| Idiopathic Parkinson Disease | Sensitivity | 91.2% | 96.8% | 91.9% |
|  | Specificity | 90.0% | 91.2% | 99.9% |
|  | PPV | 95.1% | 95.2% | 99.9% |
|  | NPV | 82.0% | 93.9% | 87.2% |
| Multiple System Atrophy | Sensitivity | 90.9% | 95.0% | 95.0% |
|  | Specificity | 95.8% | 98.7% | 98.7% |
|  | PPV | 80.0% | 95.0% | 95.0% |
|  | NPV | 98.2% | 98.7% | 98.7% |
| Progressive Supranuclear Palsy | Sensitivity | 73.8% | 71.4% | 99.9% |
|  | Specificity | 94.5% | 96.3% | 94.0% |
|  | PPV | 70.5% | 76.9% | 73.7% |
|  | NPV | 95.3% | 95.2% | 99.9% |

^1^ The statistics of Overall summarizes the accuracy of all the 280 patients on initial [^11^C]CFT PET scans.

^2^ The statistics of Baseline summarizes the accuracy of 96 patients (with both initial and repeated PET scans) on the initial [^11^C]CFT PET scans.

^3^ The statistics of Follow-up summarizes the accuracy of 96 patients (with both initial and repeated PET scans) on the repeated [^11^C]CFT PET scans.

PPV and NPV represent positive predictive value and negative predictive value.

**Supplementary Table 5** Statistical testing of AUC differences of the DAT-Net for the differential diagnosis of the parkinsonian disorders evaluated on the training cohort. The evaluation is performed using cross-validation during the development of the DAT-Net.

|  |  | P-value |
| --- | --- | --- |
| Idiopathic Parkinson Disease (IPD) | Overall vs Long | 0.888 |
|  | Overall vs Short | 0.887 |
|  | Long vs Short | 0.809 |
| Multiple System Atrophy (MSA) | Overall vs Long | 0.651 |
|  | Overall vs Short | 0.772 |
|  | Long vs Short | 0.535 |
| Progressive Supranuclear Palsy (PSP) | Overall vs Long | 0.626 |
|  | Overall vs Short | 0.588 |
|  | Long vs Short | 0.381 |

**Supplementary Table 6** The statistical significance of the AUC obtained by the DAT-Net for the differential diagnosis of the parkinsonian disorders evaluated on the training cohort. The evaluation is performed using cross-validation during the development of the DAT-Net.

|  | Overall | Short Symptom Durations  (≤ 2 years) | Long Symptom Durations  (> 2 years) |
| --- | --- | --- | --- |
| IPD | 0.953, P<0.0001 | 0.931, P<0.0001 | 0.950, P<0.0001 |
| MSA | 0.948, P<0.0001 | 0.958, P<0.0001 | 0.925, P<0.0001 |
| PSP | 0.900, P<0.0001 | 0.855, P<0.0001 | 0.921, P<0.0001 |
| NC | 0.998, P<0.0001 | / | / |

**Methodology Details**

1. Detailed Network Architecture

The detailed network architecture and parameter information are shown in the following table. (Our code is available for download at <https://github.com/Louis-YuZhao/DAT-NET.git>)

**Supplementary Table 7** the detailed network architecture and parameters of the proposed DAT-Net

| **Layer (type)** | **Output Shape** | **Param #** | **Connected to** |
| --- | --- | --- | --- |
| layer_0 (InputLayer) | (None, 96, 96, 96, 1) | 0 |  |
| layer_1 (Conv3D) | (None, 48, 48, 48, 64) | 8064 | layer_0[0][0] |
| layer_2 (InstanceNormalization) | (None, 48, 48, 48, 64) | 128 | layer_1[0][0] |
| layer_3 (LeakyReLU) | (None, 48, 48, 48, 64) | 0 | layer_2[0][0] |
| layer_4 (MaxPooling3D) | (None, 24, 24, 24, 64) | 0 | layer_3[0][0] |
| layer_5 (Conv3D) | (None, 24, 24, 24, 64) | 110656 | layer_4[0][0] |
| layer_6 (InstanceNormalization) | (None, 24, 24, 24, 64) | 128 | layer_5[0][0] |
| layer_7 (LeakyReLU) | (None, 24, 24, 24, 64) | 0 | layer_6[0][0] |
| layer_8 (SpatialDropout3D) | (None, 24, 24, 24, 64) | 0 | layer_7[0][0] |
| layer_9 (Conv3D) | (None, 24, 24, 24, 64) | 110656 | layer_8[0][0] |
| layer_10 (InstanceNormalization) | (None, 24, 24, 24, 64) | 128 | layer_9[0][0] |
| layer_11 (LeakyReLU) | (None, 24, 24, 24, 64) | 0 | layer_10[0][0] |
| layer_12 (Add) | (None, 24, 24, 24, 64) | 0 | layer_4[0][0], layer_11[0][0] |
| layer_13 (Conv3D) | (None, 12, 12, 12, 128) | 221312 | layer_12[0][0] |
| layer_14 (InstanceNormalization) | (None, 12, 12, 12, 128) | 256 | layer_13[0][0] |
| layer_15 (LeakyReLU) | (None, 12, 12, 12, 128) | 0 | layer_14[0][0] |
| layer_16 (Conv3D) | (None, 12, 12, 12, 128) | 442496 | layer_15[0][0] |
| layer_17 (InstanceNormalization) | (None, 12, 12, 12, 128) | 256 | layer_16[0][0] |
| layer_18 (LeakyReLU) | (None, 12, 12, 12, 128) | 0 | layer_17[0][0] |
| layer_19 (SpatialDropout3D) | (None, 12, 12, 12, 128) | 0 | layer_18[0][0] |
| layer_20 (Conv3D) | (None, 12, 12, 12, 128) | 442496 | layer_19[0][0] |
| layer_21 (InstanceNormalization) | (None, 12, 12, 12, 128) | 256 | layer_20[0][0] |
| layer_22 (LeakyReLU) | (None, 12, 12, 12, 128) | 0 | layer_21[0][0] |
| layer_23 (Add) | (None, 12, 12, 12, 128) | 0 | layer_15[0][0], layer_22[0][0] |
| layer_24 (Conv3D) | (None, 6, 6, 6, 256) | 884992 | layer_23[0][0] |
| layer_25 (InstanceNormalization) | (None, 6, 6, 6, 256) | 512 | layer_24[0][0] |
| layer_26 (LeakyReLU) | (None, 6, 6, 6, 256) | 0 | layer_25[0][0] |
| layer_27 (Conv3D) | (None, 6, 6, 6, 256) | 1769728 | layer_26[0][0] |
| layer_28 (InstanceNormalization) | (None, 6, 6, 6, 256) | 512 | layer_27[0][0] |
| layer_29 (LeakyReLU) | (None, 6, 6, 6, 256) | 0 | layer_28[0][0] |
| layer_30 (SpatialDropout3D) | (None, 6, 6, 6, 256) | 0 | layer_29[0][0] |
| layer_31 (Conv3D) | (None, 6, 6, 6, 256) | 1769728 | layer_30[0][0] |
| layer_32 (InstanceNormalization) | (None, 6, 6, 6, 256) | 512 | layer_31[0][0] |
| layer_33 (LeakyReLU) | (None, 6, 6, 6, 256) | 0 | layer_32[0][0] |
| layer_34 (Add) | (None, 6, 6, 6, 256) | 0 | layer_26[0][0], layer_33[0][0] |
| layer_35 (Conv3D) | (None, 3, 3, 3, 512) | 3539456 | layer_34[0][0] |
| layer_36 (InstanceNormalization) | (None, 3, 3, 3, 512) | 1024 | layer_35[0][0] |
| layer_37 (LeakyReLU) | (None, 3, 3, 3, 512) | 0 | layer_36[0][0] |
| layer_38 (Conv3D) | (None, 3, 3, 3, 512) | 7078400 | layer_37[0][0] |
| layer_39 (InstanceNormalization) | (None, 3, 3, 3, 512) | 1024 | layer_38[0][0] |
| layer_40 (LeakyReLU) | (None, 3, 3, 3, 512) | 0 | layer_39[0][0] |
| layer_41 (SpatialDropout3D) | (None, 3, 3, 3, 512) | 0 | layer_40[0][0] |
| layer_42 (Conv3D) | (None, 3, 3, 3, 512) | 7078400 | layer_41[0][0] |
| layer_43 (InstanceNormalization) | (None, 3, 3, 3, 512) | 1024 | layer_42[0][0] |
| layer_44 (LeakyReLU) | (None, 3, 3, 3, 512) | 0 | layer_43[0][0] |
| layer_45 (Add) | (None, 3, 3, 3, 512) | 0 | layer_37[0][0], layer_44[0][0] |
| layer_46 (Conv3D) | (None, 3, 3, 3, 512) | 262656 | layer_45[0][0] |
| layer_47 (InstanceNormalization) | (None, 3, 3, 3, 512) | 1024 | layer_46[0][0] |
| layer_48 (LeakyReLU) | (None, 3, 3, 3, 512) | 0 | layer_47[0][0] |
| layer_49 (GlobalAveragePooling) | (None, 512) | 0 | layer_48[0][0] |
| layer_50 (Dense) | (None, 4) | 2052 | layer_49[0][0] |
| Total params: 23,727,876 | | | |
| Trainable params: 23,727,876 | | | |
| Non-trainable params: 0 | | | |

1. Pre-training details

In our work, the “clinically possible” diagnoses are diagnoses without return visit and over-two-years clinical follow-up, therefore are not as confident as the “clinically definite” and “clinically confirmative” diagnoses. To leverage more samples to contribute to the development of the proposed DAT-Net, we utilized the patients with the clinically possible diagnosis as the pre-training cohort and in the pre-training stage, we warm up the network and did not allow the network (DAT-Net) to be fully converged to prevent over-fitting the network with possible incorrect diagnoses. Based on the pre-trained weights, we then fine-tune the DAT-Net on the training cohort and allow it to be fully converged (We utilized the data augmentation and early-stopping strategies to prevent over-fitting). More importantly, during the fine-tuning stage, we only re-used the pre-trained weights of early convolutional layers before the global average pooling layer (working for extracting informative features in the DAT-Net) and did not utilize the pre-trained weights of the final multilayer perceptron head (MLP, working for mapping the learnt features to final diagnoses in the DAT-Net) to alleviate the impact of incorrect labels.

1. **Data augmentation**

We performed data augmentation methods including (1) Besides using FWHM=10, applying different 3D Gaussian filters (FWHM=5, and FWHM=8) to smoothen the normalized PET images during pre-processing to generate more training samples (The PET scans smoothed with FWHM=5, and FWHM=8 were only utilized during the training stage to enlarger the training dataset.). (2) Randomly adding Gaussian noise with average (equal to 0) and a random variance ranging from 0 to 0.1 (We discussed with domain experts to make sure the reasonableness of the augmenting strategy in clinical practice.).

1. **The Full-gradient Saliency Map**

The full-gradient saliency map method is proposed in [1], which considers both the input importance indicating the contribution of individual input voxels and neuron importance reflecting the contribution of groups of voxels with specific structural information, which is sharper and more tightly confined to object regions compared to other existing methods such as Integrated gradient [2], Smooth-grad [3], Grad-CAM [4], etc. [1]. The full-gradients achieve high performance by simultaneously satisfying both notions of local and global importance [1]. The author offered a user-friendly code, and we utilized it to implement the full-gradient saliency map. The code is available at https://github.com/idiap/fullgrad-saliency.

1. **The Most Saliency Regions Selection**

The detailed steps for selecting the top salient regions are as follows

Assuming the salient map is denoted as $Xs$, the first step is to calculate the absolute value elementwise, i.e,

${X^{'}}_{s}=ABS(X_{s})$. (1)

Then we use the MinMax normalization to normalize $X^{'}s$as follows:

${X^{''}}_{s}=MinMax\left( {X^{'}}_{s} \right)={{{(X}^{'}}_{s}-Min({X^{'}}_{s}))}/{(Max\left( {X^{'}}_{s} \right)-Min({X^{'}}_{s}))}$ (2)

Finally, we choose the top T salient regions as the included regions.

$X_{include}\left( x \right)=\left\{ \begin{aligned} 1, if {X^{''}}_{s}\left( x \right) T \\ 0, if {X^{''}}_{s}\left( x \right)<T \end{aligned} \right.$ (3)

where $x$ denotes the voxel in the 3D volume, and T is the threshold (for instance T=30% in our case).

**Reference**

1. François SSA. Full-Gradient Representation for Neural Network Visualization. Advances in Neural Information Processing Systems (NeurIPS). 2019.

2. Simonyan K, Vedaldi A, Zisserman A. Deep inside Convolutional Networks: Visualising image classification models and saliency maps [Internet]. arXiv [cs.CV]. 2013. Available from: http://arxiv.org/abs/1312.6034

3. Smilkov D, Thorat N, Kim B, Viégas F, Wattenberg M. SmoothGrad: removing noise by adding noise [Internet]. arXiv [cs.LG]. 2017. Available from: http://arxiv.org/abs/1706.03825

4. Selvaraju RR, Cogswell M, Das A, Vedantam R, Parikh D, Batra D. Grad-CAM: Visual explanations from deep networks via gradient-based localization. Int J Comput Vis. Springer Science and Business Media LLC; 2020;128:336–59.
